# Shape-Based Virtual Screening of a Billion-Compound Library Identifies Mycobacterial Lipoamide Dehydrogenase Inhibitors

**DOI:** 10.1021/acsbiomedchemau.3c00046

**Published:** 2023-09-08

**Authors:** Mayako Michino, Alexandre Beautrait, Nicholas A. Boyles, Aparna Nadupalli, Alexey Dementiev, Shan Sun, John Ginn, Leigh Baxt, Robert Suto, Ruslana Bryk, Steven V. Jerome, David J. Huggins, Jeremie Vendome

**Affiliations:** † Sanders Tri-Institutional Therapeutics Discovery Institute, 1230 York Avenue, Box 122, New York, New York 10065, United States; ‡ Schrödinger, Inc., 1540 Broadway, 24th Floor, New York, New York 10036, United States; § Schrödinger, Inc., 12 Michigan Dr., Natick, Massachusetts 01760, United States; ∥ Department of Microbiology and Immunology, Weill Cornell Medicine, New York, New York 10065, United States; ⊥ Department of Physiology and Biophysics, Weill Cornell Medicine, New York, New York 10021, United States

**Keywords:** virtual screening, tuberculosis, mycobacteria, lipoamide dehydrogenase, inhibitor

## Abstract

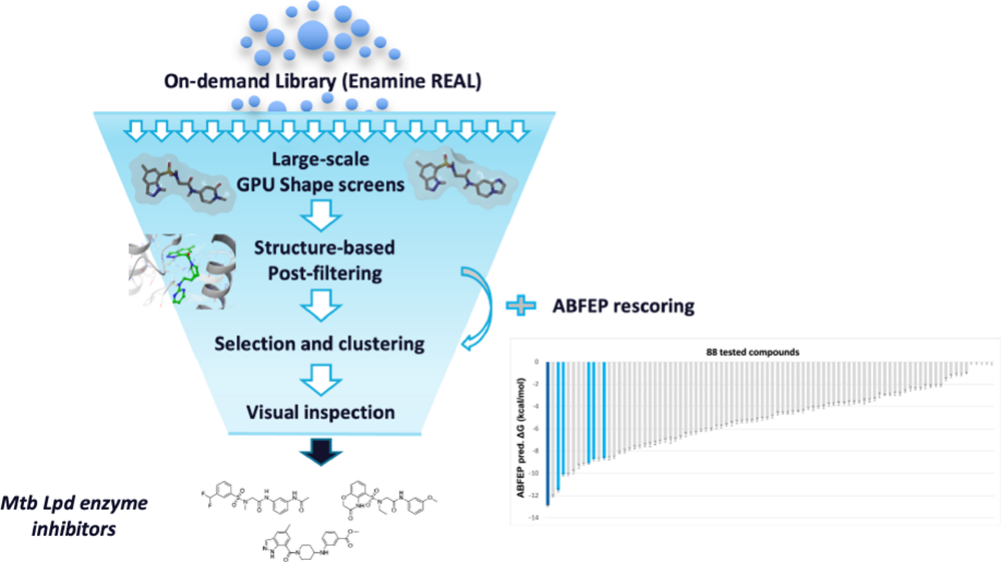

Lpd (lipoamide dehydrogenase) in *Mycobacterium tuberculosis* (Mtb) is required for virulence and is a genetically validated tuberculosis (TB) target. Numerous screens have been performed over the last decade, yet only two inhibitor series have been identified. Recent advances in large-scale virtual screening methods combined with make-on-demand compound libraries have shown the potential for finding novel hits. In this study, the Enamine REAL library consisting of ∼1.12 billion compounds was efficiently screened using the GPU Shape screen method against Mtb Lpd to find additional chemical matter that would expand on the known sulfonamide inhibitor series. We identified six new inhibitors with IC_50_ in the range of 5–100 μM. While these compounds remained chemically close to the already known sulfonamide series inhibitors, some diversity was found in the cores of the hits. The two most potent hits were further validated by one-step potency optimization to submicromolar levels. The co-crystal structure of optimized analogue **TDI-13537** provided new insights into the potency determinants of the series.

## Introduction

Lipoamide dehydrogenase (Lpd) is an oxidoreductase component of the four major metabolic complexes inside most living cells. It generates NADH during oxidative decarboxylation of pyruvate, alpha-ketoglutarate, branched chain ketoacids, and glycine contributing to the production of high energy molecules and metabolic intermediates.^1−4^ While the metabolic function of Lpd is ubiquitous, Lpd fulfills an additional antioxidant function in mycobacteria, including *Mycobacterium tuberculosis* (Mtb), a causative agent of tuberculosis (TB), and provides electrons for the detoxification of reactive oxygen and nitrogen species.^5^ TB remains a leading cause of death from a single bacterial infection worldwide.^6,7^ Rapidly rising drug resistance to current TB antibiotics calls for the development of new therapeutic strategies with expansion of the target space. Lpd in Mtb is required for virulence and persistence in mice and is a genetically validated TB target.^8^

**Figure 1:**
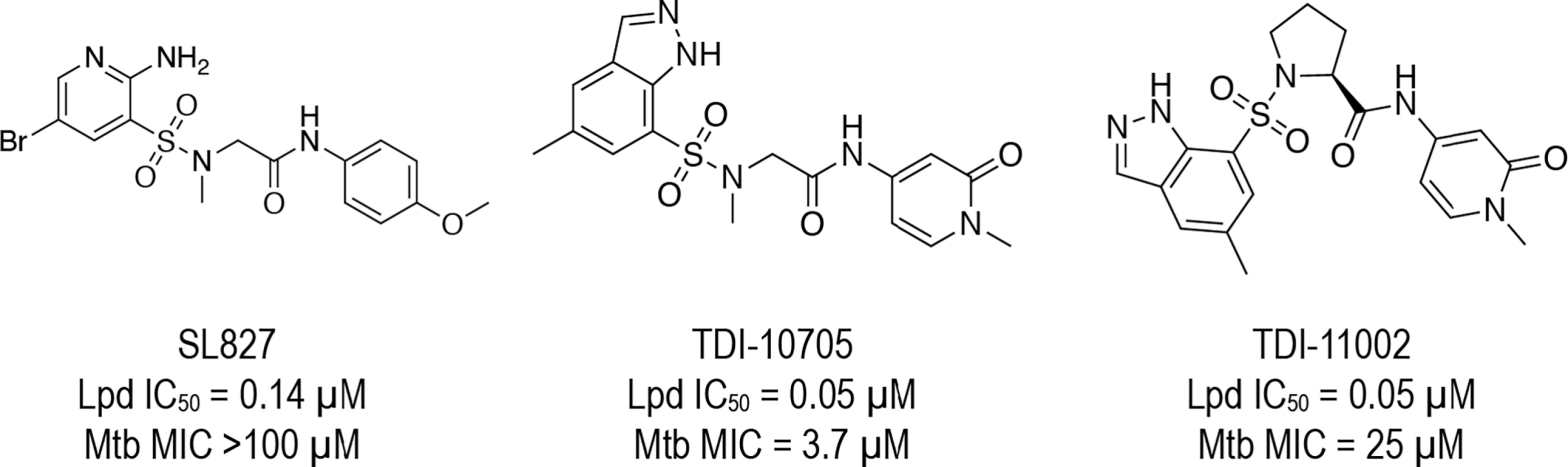
Representative Lpd inhibitors from the sulfonamide series.

While Lpd is an attractive therapeutic target for TB, finding small molecule chemical matter with inhibitory activity has been extremely challenging. Numerous experimental high-throughput screens have been performed over the last decade, yet only a few inhibitor series have been identified (Table 1). We have used a high-throughput enzymatic assay to screen over 2.5 million compounds from commercial and academic combinatorial chemical libraries (ChemDiv, Cerep, Prestwick, Albany Molecular, Academia Sinica, Broad Institute, and others) as well as natural product libraries (AnalytiCon and Eskitis Institute Nature Bank). Across all these high-throughput screening (HTS) campaigns, we identified only two inhibitor scaffolds that met the following criteria: potent inhibition of Lpd in the DTNB assay, inhibition of the recombinant Mtb PDH complex and the native PDH complex in Mtb lysates, and high selectivity against both thioredoxin reductase (an NADPH-dependent oxidoreductase) and human Lpd. These were the triazaspirodimethoxybenzoyl series and the sulfonamide series.^9,10^ Of these two inhibitor series, the sulfonamide series emerged as the chemotype that could be optimized to demonstrate efficacy in inhibiting the growth of Mtb (Figure 1).^11^

**Table 1:** Prior Screening Efforts to Identify Lpd Inhibitors

library	number of compounds screened	number of hits	lead inhibitor identified
ChemDiv	18,100	14 (non-specific)	none
deCODE Iceland	100,000	5–10 (non-specific)	none
TBSGC virtual screen	1,750,000	38	none
Scripps/UCLA	N/A	N/A	none
ChemBridge, Spectrum, Cerep	14,000	10 (general toxins)	none
Albany Molecular Research	50,000	50	triazaspirodimethoxy-benzoyl Cmp 5 (IC_50_ = 0.9 μM, binds in NADH pocket)^10^
AnalytiCon	2,079	28	none
Academia Sinica	2,000,000	283	sulfonamide SL827 (IC_50_ = 0.14 μM, binds in lipoamide pocket)^9^
University of Kansas	3,310	0	none
Boston University	1,258	0	none
Broad Institute	84,000	3	none
Eskitis Institute Nature Bank	202,268	175	none

**Figure 2:**
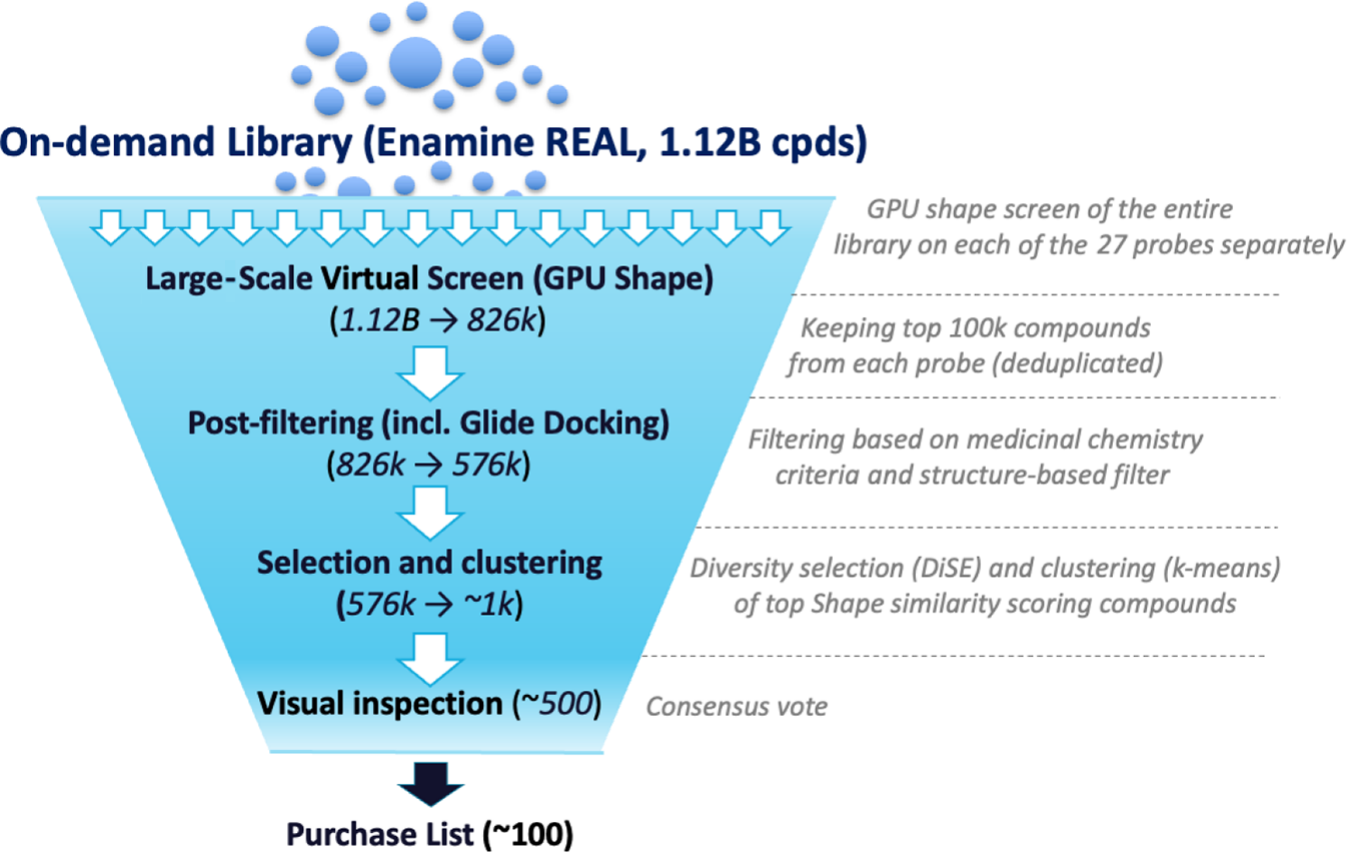
Summary workflow of the GPU Shape ultra-large virtual screening campaign.

Given that Mtb Lpd is a valuable but challenging drug target with an array of in vitro enzymatic and bacteria growth assays available, we propose it as a good test case to validate advanced hit-finding methods. Recent advances in large-scale virtual screening methods combined with make-on-demand compound libraries consisting of more than 100 million compounds have shown the potential to lead to more diverse hits and higher hit rates.^12,13^ The ultra-large virtual libraries such as Enamine REAL,^14^ Mcule ultimate,^15^ and WuXi GalaXi^16^ continue to expand rapidly and now comprise over hundreds of million to tens of billion compounds. However, it is worth noting that this only represents a small fraction of the entire chemical space, which is estimated to contain 10^60^ drug-like molecules.^17^ With increasing library size, the computational resource required to perform virtual screening becomes the bottleneck. Several approaches have been reported in the literature to overcome the computational cost. One such approach is a GPU-based cloud computing platform in combination with a fast 3D ligand shape-based method.^18^ Another approach is to use machine-learning enhanced molecular docking to increase throughput over traditional docking.^19^ Most recently reported is the modular synthon-based approach to perform hierarchical structure-based screening.^20^ In this study, we performed an ultra-large virtual screen against Mtb Lpd using a ligand-based method called GPU Shape, implemented in the Schrödinger suite^21^ (Figure 2). In the GPU Shape screen method, compounds in the library are flexibly aligned to the bioactive conformation of known actives provided as input, or probes, and a shape similarity score is computed as a hard sphere overlap.^21^ The GPU Shape program can compute up to ∼105,000 conformers/s and does not require a high-memory server, making it feasible to screen ultra-large compound libraries. The objective of this virtual screen campaign for Mtb Lpd was to evaluate whether an ultra-large-scale screen would lead to additional chemical matter to expand on the known sulfonamide inhibitor series.

## Results

### GPU Shape Ultra-Large Virtual Screen Hits

**Figure 3:**
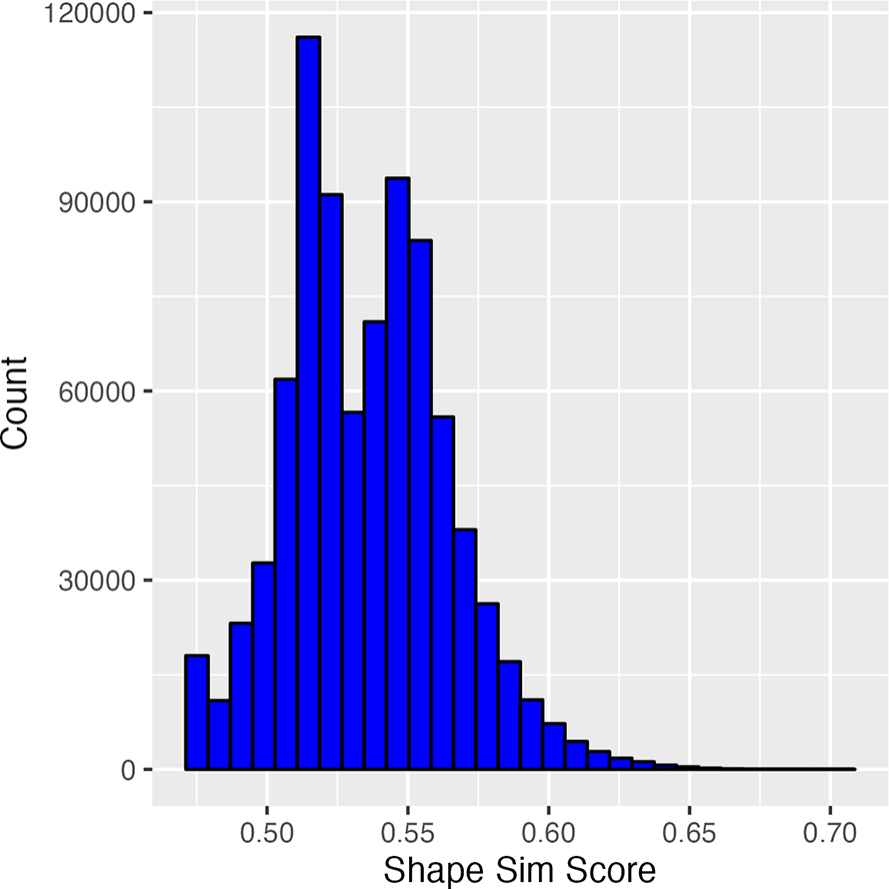
Cumulative GPU Shape similarity score distribution of the top 100 k hits (per probe) from the 27 probes.

The Enamine REAL library, consisting of ∼1.12 billion compounds at the time of this study, was screened using the GPU Shape methodology implemented by Schrödinger.^21^ Briefly, we selected 27 compounds known to inhibit Lpd with IC_50_ < 1.0 μM in our Lpd enzyme assay (Table S1) and used each of them as a separate probe for the GPU Shape screen. For each probe, we kept the 100 k top-scoring compounds out of the GPU Shape screen (∼0.01% of the library) and finally obtained 826 k unique compounds, with shape similarity scores ranging from ∼0.47 to 0.70 (Figure 3). As several X-ray structures are available for Lpd, including complexes with some of our probes (PDB: 4M52 and 7KMY), we decided to triage the GPU Shape top-scoring compounds with a structure-based post-filtering step by docking the 826 k compounds in the lipoamide pocket using Glide SP. Importantly, since a significant advantage of ligand-based screening approaches such as GPU Shape is to avoid strong dependence on a particular structural conformation of the target, docking was only meant to filter out compounds with low likelihood of fitting into the targeted pocket (compounds with docking scores above −5 kcal/mol were filtered out, leaving 576 k compounds). From the triaged set, compounds with either a high shape similarity score (Shape Sim score > 0.63) or high docking score (docking score < −9.5) were selected as the best scoring compounds, totaling 1092 hits (Figure S1, see the Experimental Section for more details). These best scoring compounds were then clustered and visually inspected to derive 103 diverse and representative hits. The visual inspection focused on evaluating features such as protein–ligand interactions in the binding pocket, conformational strain of compound poses, novelty, and medicinal chemistry progressibility of compounds. Of the 103 compounds selected for purchase, 88 compounds were synthesized at Enamine.

**Figure 4:**
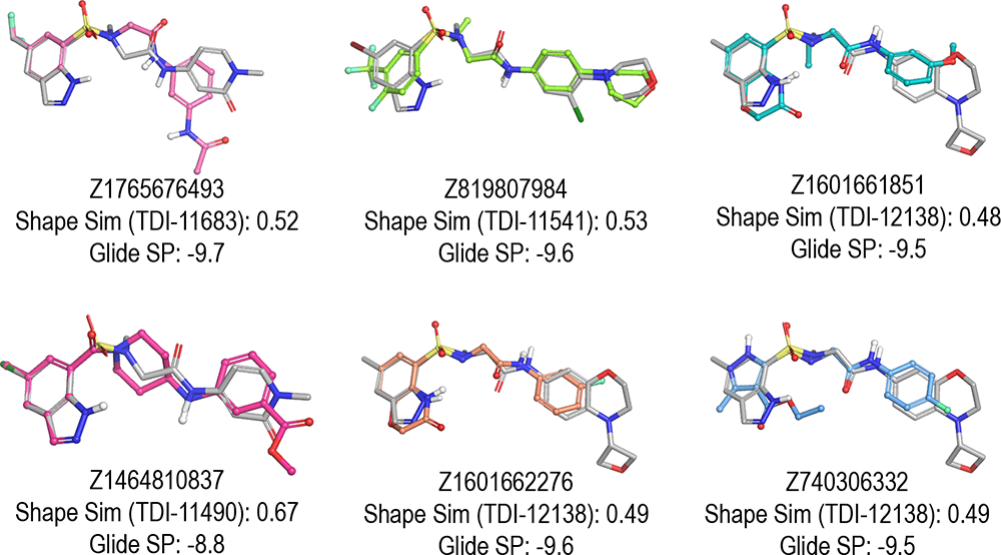
GPU Shape screen hit superposition with the probe (reference probe shown with gray carbon atoms).

The 88 purchased compounds were tested in the Lpd enzyme assay first at 10 μM and then in dose response for compounds with an inhibitory activity greater than ∼10%. We found six hits in total, corresponding to a hit rate of ∼7% (Table 2). Two hit compounds have an IC_50_ of 5 μM, two others have an IC_50_ of 30 μM, and the last two hits are weaker with an IC_50_ of around 100 μM (Table 2 and Figure 4).

**Table 2:**
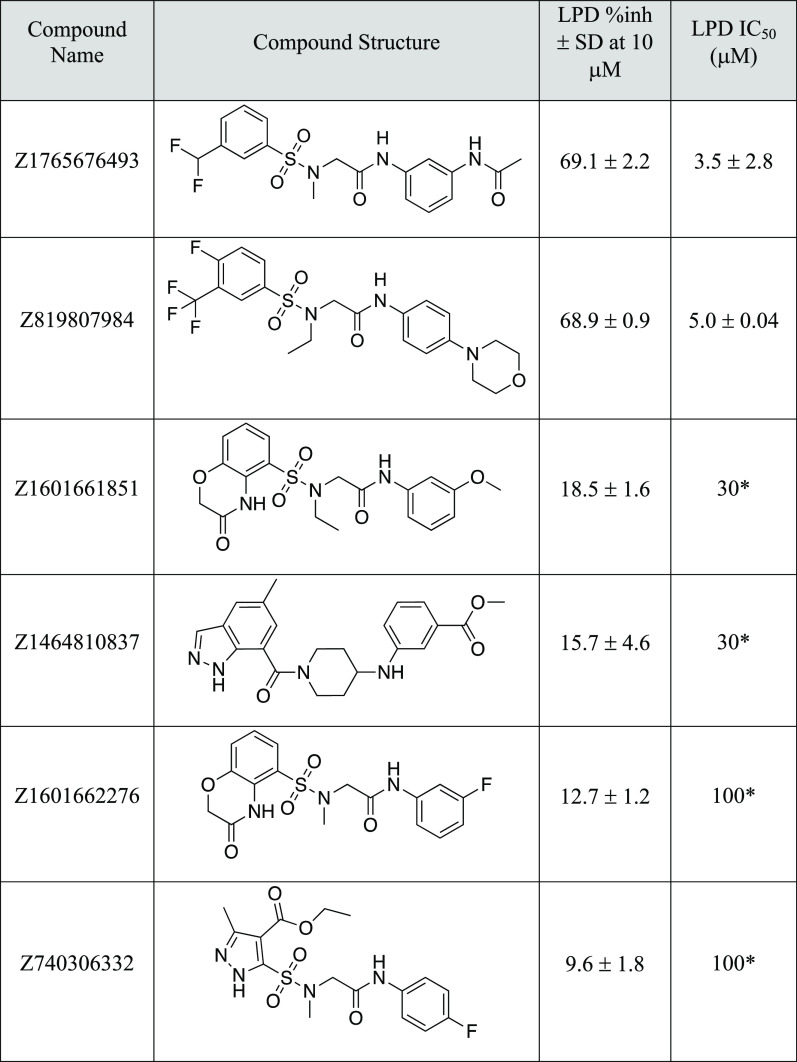
GPU Shape Screen Hits and Their Lpd Inhibitory Activities

a IC_50_ value from one replicate.

The six hits identified have a clear similarity to the 27 probes used in our GPU Shape screen, but they also displayed some variations. The 27 probes shared a common scaffold: a substituted indazole core with the sulfonamide linkage. The indazole core was found in one hit but replaced by a substituted phenyl or a substituted pyrazole or benzoxazinone in the other hits. Also, five out of the six hits had a sulfonamide linkage, but one hit had an amide, which replaced the sulfonamide in the scaffold (Z1464810837). More chemical diversity was present in the top GPU Shape scoring compounds as well as in the 88 compounds selected for experimental testing (Figure S2). However, none of the more diverse compounds tested was active, confirming the very narrow chemical space for achieving activity in Mtb Lpd.

### False Positive Discrimination by ABFEP Rescoring

**Figure 5:**
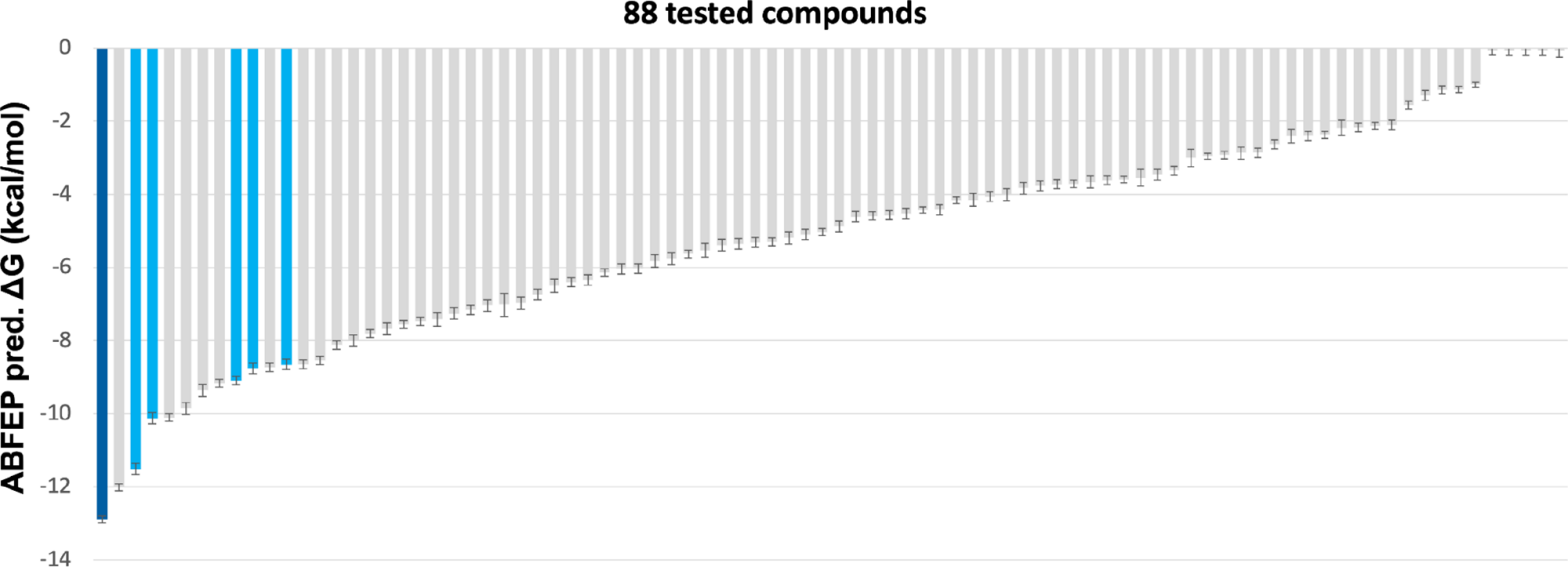
Retrospective free energy of binding predictions by ABFEP. Retrospective absolute binding FEP+ predictions on the 88 experimentally tested compounds. The six validated hits are highlighted in blue or navy blue (for compound Z1765676493).

Given the availability of Lpd crystal structures and the relatively good docking scores of the 88 compounds experimentally tested, we decided to test the ability of rescoring using absolute protein–ligand binding free energy calculation by the free energy perturbation method^22−27^ (ABFEP) to discriminate false positives (compounds with good GPU Shape and/or docking score but inactive experimentally) from true positives (experimentally active compounds). The ABFEP method implemented in the Schrödinger FEP+ suite has been previously shown to accurately predict absolute binding free energies for sets of diverse compounds and has recently been applied as a final rescoring method in virtual screening campaigns to filter out false positives and improve hit rates.^28^ We used this ABFEP method to retrospectively predict the binding affinity of the 88 compounds experimentally tested. While the 88 compounds could not be effectively triaged by either GPU shape or docking score (Figure S3), ABFEP rescoring resulted in a dramatic enrichment of true positive hits at the very top as the six confirmed hits are ranked within the top 12 ABFEP scoring compounds (corresponding to a 50% hit rate within the top 12 compounds) (Figure 5).

### One-Step Potency Optimization of the Best Hits

To further validate the most potent GPU Shape screen hits, we tested whether the structure–activity relationships (SAR) in the known sulfonamide inhibitor series could be transferred to the virtual screen hits, given their common sulfonamide linkage scaffold, to improve the potency. Specifically, we noted from the pre-existing SAR that adding a methyl group at the 5-position of the indazole or amino-pyridine core improved potency by 35- to 90-fold (Table S2). Therefore, we synthesized methylated analogues of Z1765676493 and Z819807984 at the analogous position, leading to **1** (**TDI-13537**) and **2**, respectively. Gratifyingly, the potency of both **1** (**TDI-13537**) and **2** improved by 15- to 20-fold to sub-micromolar levels in one-step optimization (Table 3).

**Table 3:**
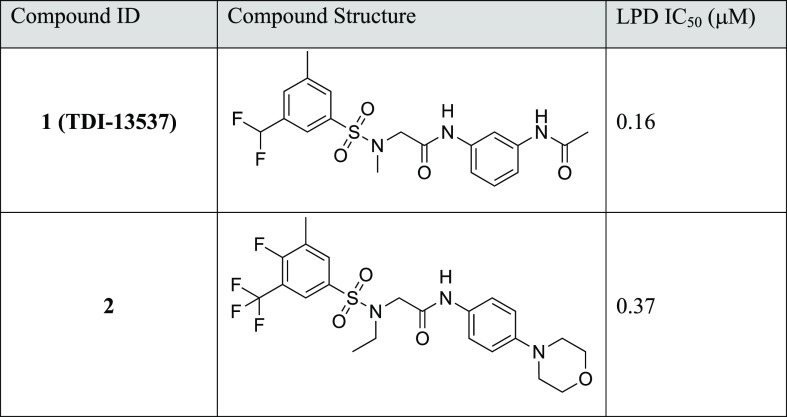
One-Step Optimized Analogues of GPU Shape Screen Hits

Addition of a methyl group to a phenyl ring is a common transformation in the hit-to-lead stage of drug discovery. Thus, the GPU Shape screen hits Z1765676493 and Z819807984 were demonstrated to be progressible Mtb Lpd hits.

### Biophysical Profiling and Structure of the Lpd-**TDI-13537** Co-Crystal

To complement the enzymatic assay and provide additional profiling of **TDI-13537**, the most potent molecule derived from the screen, we evaluated its binding affinity for Lpd and its binding kinetics using surface plasmon resonance (SPR). The binding affinity of **TDI-13537** is consistent with its enzymatic potency (*K*_D_ = 0.241 μM compared to Lpd IC_50_ = 0.16 μM) (Table 4 and Figure S4). The measurement of association and dissociation rates indicated that **TDI-13537** has a fast on–off binding profile. Slow dissociation has been observed for some sulfonamide series inhibitors and has been demonstrated to drive the whole-cell activity in Mtb.^11^ Consistently, **TDI-13537** did not show inhibition of Mtb bacterial growth in the MIC assay (data not shown).

**Table 4:**
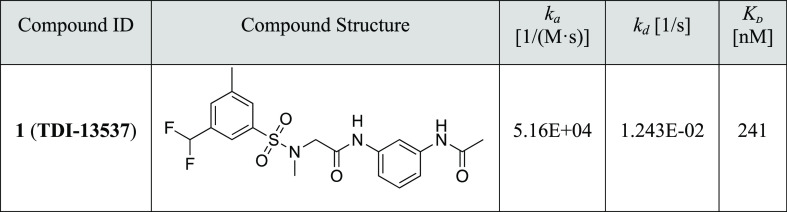
SPR Binding Affinity of **TDI-13537**

**Figure 6:**
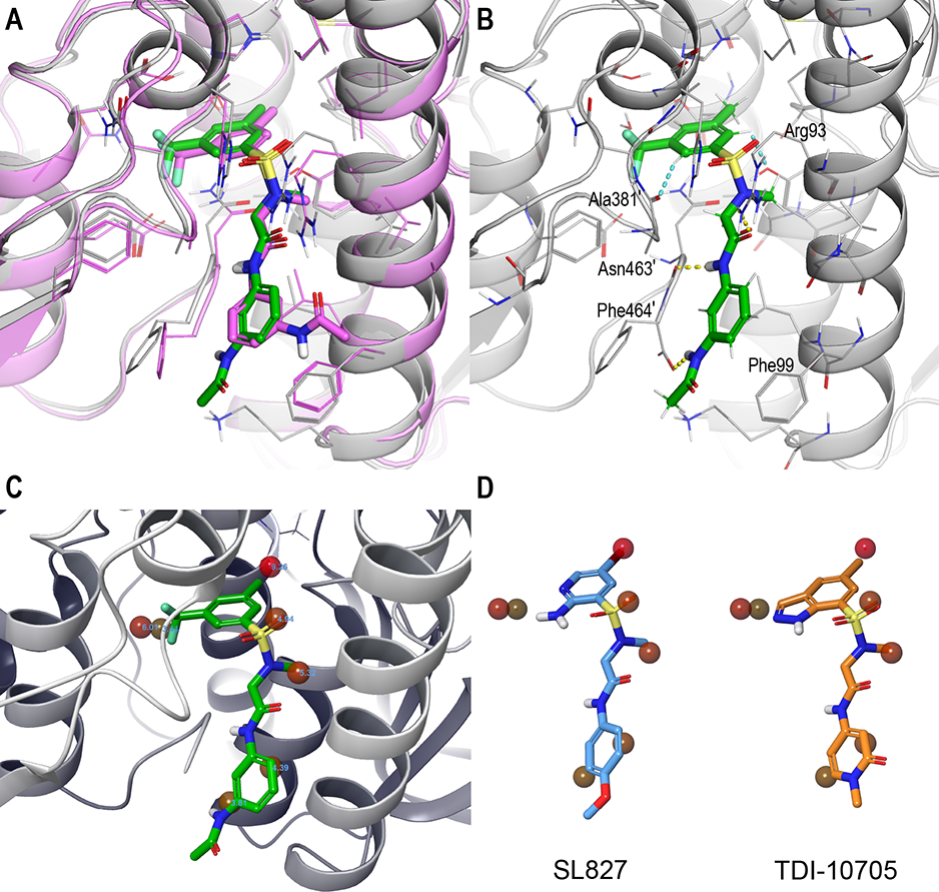
Co-crystal structure of Lpd with **TDI-13537**. (A) Superposition of the co-crystal structure (protein in gray and ligand **TDI-13537** in green sticks) with predicted docking pose (in magenta), (B) protein–ligand interactions of **TDI-13537** (canonical hydrogen bonds in yellow dotted lines and aromatic CH–O hydrogen bonds in cyan dotted lines), (C) displacement of WaterMap predicted high-energy waters by ligand **TDI-13537** (spheres with a shade of red and labels corresponding to Δ*G* free energy of the hydration site), and (D) co-crystal ligand pose of known actives SL827 (in blue sticks; PDB: 4M52) and TDI-10705 (in orange sticks; PDB: 7KMY), shown in the same orientation as **TDI-13537** in panel (C) (protein removed for clarity and the displaced WaterMap high-energy waters from panel (C)).

Intrigued by the absence of a hydrogen bond donor on the novel core of the two best hits and their corresponding derivatives, which we thought was necessary for potency within the sulfonamide series based on previous SAR, we then characterized the binding mode of **TDI-13537** in Lpd by X-ray crystallography. The binding mode of **TDI-13537** in the co-crystal structure superimposed very well with the predicted docked pose, including for the asymmetrical substituents on the core moiety (Figure 6A). The only inaccuracy in the docked pose was toward the solvent-exposed region of the compound, where the orientation of the terminal acetamide group was flipped to the other side of the phenyl ring. The compound occupies the lipoamide binding site and forms all the expected protein–ligand interactions (Figure 6B). The sulfonamide moiety orients the central amide’s carbonyl group to form a hydrogen bond with the side chain of Arg93, which was previously found to be a critical residue for Mtb over human selectivity.^9^ The terminal phenyl moiety stacks with the Phe99 side chain, and the acetamide-NH substituent interacts with the Phe464′ C-terminal backbone carboxyl group. Interestingly, while **TDI-13537** lacks an NH hydrogen bond donor that is conserved in the core of known sulfonamide inhibitor series (such as the aminopyridine in SL827 and indazole in **TDI-10705**), the phenyl-CH ortho to the CHF2 substituent is oriented to potentially make a weak non-canonical aromatic CH–O hydrogen bond interaction^29,30^ with the Ala381′ backbone carbonyl.

To better understand the potency increase in **TDI-13537** compared to the des-methyl screen hit Z1765676493, we used WaterMap^31,32^ to calculate the location and thermodynamic properties of hydration sites in the lipoamide pocket. In the absence of ligands (apo pocket), several high-energy hydration sites are identified in the pocket, many of them overlapping with the position of the **TDI-13537** in the X-ray structure and therefore contributing to the binding energetics of the ligand to the pocket (Figure 6C). The additional methyl group of **TDI-13537**, in particular, displaces a weakly bound water molecule calculated to be energetically less stable than in the bulk solvent (Δ*G* = 9.3 kcal/mol), and that would not be displaced in the absence of the methyl group, thus explaining the sharp potency gain (Figure 6C). The displacement of the same weakly bound water is also likely to explain the previously observed, but unexplained so far, effect of a similar methylation on different cores. Notably, **TDI-13537** with the novel CHF2-substituted phenyl core has comparable potency to known actives with different cores such as the indazole or amino-pyridine core but without the canonical hydrogen bond donor previously thought to be necessary (Figure 6D).

## Discussion and Conclusions

The study presented here undertook a double challenge: first, identifying new inhibitors of Mtb Lpd, which has repeatedly proven to be extremely difficult in the past regardless of the approach, and second, doing so *via* a ligand-based virtual screening approach, GPU Shape screen, applied at an ultra-large scale. Lessons were learned in tackling both challenges.

From the perspective of hit discovery for Mtb Lpd, while the virtual screen effort successfully identified six new inhibitors, these compounds remained chemically close to the already known sulfonamide series inhibitors, confirming once again the extreme difficulty to identify novel active chemical matter against this target and suggesting a particularly narrow chemical space for Mtb Lpd inhibitors. Despite this challenge, some diversity in the cores of the hits identified point to some level of flexibility in that region. Furthermore, the detailed analysis of **TDI-13537**, a potent inhibitor with a novel core derived from the GPU Shape screen, brought new insights into the potency determinants of the entire series. Notably, this revealed the critical importance of the displacement of a specific weakly bound water molecule deep in the lipoamide pocket and the lesser relative importance of a hydrogen bond thought to be necessary for potency until now.

From the perspective of virtual screening methodology, the study confirms that the GPU Shape method used here is very well adapted for the screening of ultra-large libraries consisting of over a billion compounds. Each of the 27 separate screens of the ∼1.12 billion compound library (one screen per probe) required 156 GPU hours, and the entirety of the 27 GPU Shape screens was completed within 19.5 h (wall clock time) on 216 GPUs. A noticeable advantage of the GPU Shape method implemented in the Schrödinger suite is that it does not require a high-memory server since the screening library is prepared prior to the screening. This requires a one-time pre-processing, as described in the Experimental Section.

Independently of their actual activity on Lpd, it is noticeable that the GPU Shape top-scoring compounds, while generally, and expectedly, reminiscent of the sulfonamide scaffold, show a certain level of diversity as compared to the set of probes (Figure S2). Of course, one way to expand the diversity of the top-scoring compounds yielded by a GPU Shape screen is to increase the number of probes and more importantly increase their chemical diversity as much as possible, which is a specific challenge of the system we worked on in this study, as noted previously.

Finally, the availability of good quality X-ray structures for the target allowed us to retrospectively test the discriminating power of a structure-based approach consisting of docking followed by free energy calculation for rescoring using absolute binding free energy perturbation (ABFEP), a method that allows the highly accurate FEP+ predictions to be applied to sets of diverse compounds.^28^ Our results strikingly confirmed the ability of ABFEP to discriminate false positives from true positives, theoretically improving the early hit enrichment from ∼7% for the actual screen (or ∼18% for docking as it rank-ordered the six confirmed hits within the top 33 compounds) to 50%. Notably, the rank order of the best-scoring hit compound, Z1765676493, did not change and the enrichment was maintained when ABFEP calculation was performed with the flipped pose of the terminal acetamide group, similarly to the pose in the co-crystal structure of **TDI-13537** (Δ*G* = −12.2 ± 0.1 kcal/mol for the corrected pose and Δ*G* = −12.9 ± 0.1 kcal/mol for the docked pose). Altogether, this suggests a huge promise for using ABFEP prospectively in the final rescoring step of a virtual screening workflow whenever the target is structurally enabled. However, as screening libraries continue to expand and the scale at which ABFEP is applied becomes larger, the computational cost of performing ABFEP calculations presents another challenge. Additional methodological advances will be needed to address such challenges. One solution we are currently exploring is to combine ABFEP calculations with active learning methods, which may lead to considerable impact in future virtual screening campaigns.

## Experimental Section

### Preparation of the Compound Library

The Enamine REAL database compound collection (version 2019q34) comprising 1.2 billion compounds was analyzed by first generating a canonical SMILES from the desalted and neutralized form of a molecule. Molecular and chemical descriptors were then generated on this form to annotate compounds into “Clean”, “Flagged”, or “Filtered” categories, where “Clean” means no structural or property warnings applied, contrary to either “Flagged” (PAINS^33^ or potentially problematic liabilities) or “Filtered” (REOS^34^ or other known liabilities that would not benefit a drug discovery project). Fragment-type compounds (MW < 110 g/mol) were filtered out. The library was prepared with Schrödinger suite LigPrep to account for all relevant tautomeric and ionization states at pH 7.4 ± 1.0 and to enumerate the stereoisomers for structures bearing stereocenters with non-explicit chirality, up to a maximum of 16. Then, the “shape_screen_gpu generate” command was used to generate 10 conformers per structure, where each structure was treated as a set of phase pharmacophore-typed atoms (default). This produced the .bin files used for the GPU Shape screen (404.bin files of 15 Gb each, for a 6 Tb disk space total).

### GPU Shape Screen

The Schrödinger suite GPU Shape program was used to screen the Enamine REAL library, with default settings (release 2020-2, Schrödinger, LLC, New York, NY). Twenty-seven known actives having biochemical potency (Lpd enzyme inhibition IC_50_ < 1.0 μM, PDH complex inhibition IC_50_ < 1.0 μM) were selected as probes (Table S1). The bioactive conformation of the 27 probes was obtained either from co-crystal structures or by docking based on a prior sulfonamide compound-bound structure (PDB: 4M52) using the Schrödinger suite Glide SP program. The top 100 k GPU Shape hits from each probe were selected, and duplicates were removed to obtain 826 k unique top-scoring compounds. To triage these top hits, the compounds were docked into the lipoamide binding site using the Glide SP program, and those compounds with low likelihood of fit in the binding site (docking score > −5) were filtered out. From the triaged set, we selected compounds with either a high shape similarity score (1,694 hits with Shape Sim score > 0.63) or high docking score (206 hits with docking score < −9.5) and removed compounds with medicinal chemistry structural alerts (808 hits in the “Filtered” category). A total of 1,092 best-scoring compounds were then clustered by DISE^35^ and K-means^36^ clustering to derive a final set of ∼500 compounds, which was visually inspected by a team of computational and medicinal chemists. A buy list of 103 compounds was obtained by a consensus vote from the visual inspection. Of the compounds requested for purchase, 88 compounds could be synthesized at Enamine.

### Molecular Docking with Glide

The co-crystal structure of Mtb Lpd bound to the SL827 inhibitor (PDB: 4M52) was prepared using the Protein Preparation Workflow in Maestro, with the default settings (release 2020-2, Schrödinger, LLC, New York, NY). A docking grid was generated from the prepared structure, stripped of all water molecules, centered on the SL827 inhibitor, and with all default parameters. Compounds were docked using Glide SP (standard precision mode) with the generated docking grid and default parameters (release 2020-2, Schrödinger, LLC, New York, NY).

### Compound Rescoring with ABFEP

The Glide-predicted docking pose was used as starting conformation for the absolute binding FEP (ABFEP) calculation. The calculation was performed for each of the 88 experimentally tested compounds, with the default settings and as described previously,^28^ in the 2023-1 release of the Schrödinger suite.

### Themodynamics of Water in the Binding Site with WaterMap

The Lpd-**TDI-13537** complex structure was prepared using the Protein Preparation Workflow in Maestro, with the default settings (release 2023-1, Schrödinger, LLC, New York, NY). WaterMap simulations were performed on the Lpd-**TDI-13537** complex structure. Default settings were used to assess water thermodynamics in the apo state of the Lpd-**TDI-13537** binding pocket (2023-1 release of the Schrödinger suite).

### Lpd Protein Production

Recombinant protein production and purification of Mtb Lpd WT were carried out as reported previously.^9,11^

### Lpd Enzyme Assay

The Lpd enzyme assay was performed at a CRO with a previously reported protocol.^9,11^ Briefly, the Lpd activity was measured by a spectrophotometric assay with DTNB, lipoamide, and NADH. Final concentrations of components in the reaction mixture were as follows: 125 μM NADH, 125 μM DTNB, 40 μM NAD+, 75 μM lipoamide in 25 mM potassium phosphate, and 1 mM EDTA pH 7.0. Compounds were tested initially at 10 μM. Compounds showing significant inhibition at 10 μM were tested in dose–response from 100 to 0.005 μM by serial dilutions. Curves were fit, and IC_50_ values were calculated using Prism dose–response inhibition analysis (four-parameter fit).

### Chemistry

See the Supporting Information for the synthesis and characterization of **TDI-13537** and compound **2**.

### Surface Plasmon Resonance (SPR)

SPR assays were performed using a Bruker Sierra SPR-32 Pro instrument at 25 °C. The Lpd protein was attached covalently to a carboxymethylated dextran sensor (Bruker High Capacity Amine). The sensor chip was activated with a 300 s injection of 50 mM NHS/200 mM EDC at 10 μL/min. Thereafter, the Lpd protein was diluted to 1 μM in 10 mM NaOAc, pH 5.0, and injected at 10 μL/min for 180 s. After immobilization, 1 M ethanolamine was injected at 10 μL/min for 300 s to block any remaining active esters formed during the NHS/EDC injection. Control surfaces were prepared using the same protocol with the Lpd protein injection omitted.

Analytes were tested for binding in an assay buffer containing 25 mM potassium phosphate, pH 7.0, 0.05% Tween 20, 1% DMSO, and ±100 μM NADH. Compounds were diluted serially to yield a 10-point titration panel that was injected over both control and active surfaces at a flow rate of 30 μL/min with an association time of 120 s and dissociation time of 300–600 s. Assay buffer injections were included after every two analyte injections. Solvent correction standards in 0.2% DMSO intervals were run before and after the analyte samples.

Sensorgrams were analyzed using Sierra Analyzer software from Bruker with double-reference subtraction to determine the interaction parameters *k*_a_, *k*_d_, and *K*_D_. The control surface was subtracted from the active surface as the first reference. Corrected blank injections were then subtracted from the analyte injections as the double-referencing step. Binding data were analyzed for kinetic or equilibrium parameters using global analysis and a 1:1 Langmuir model to fit the data.

### Crystallization and Structure Determination

Complexes were formed between Lpd (at 200 μM in 20 mM Tris–HCl, 1 mM EDTA, pH 7.8) and **TDI-13537** (at 300 μM, added from stock solutions in 100% DMSO) at a protein/ligand ratio of 2/3 in the presence of 600 μM NADH.

Crystals of the Lpd-**TDI-13537** complex were initially obtained in sitting drops (2:2 μL) incubated at 20 °C against a reservoir solution of 0.1 M Tris–HCl, pH 8.5, 16% PEG 10,000, and 17.5% glycerol. Larger crystals grew with microseeding under similar conditions with 12–14% PEG 10,000. Crystals were cryoprotected in reservoir solution supplemented with 25% glycerol and flash frozen in liquid nitrogen.

Data were collected at beamline CMSF-ID at the Canadian Light Source, at 100 °C with an EIGER × 9 M detector at a wavelength of 0.9537 Å. A high resolution data was collected to 1.7 Å. Data were processed using the XDS suite.^37^ Data collection statistics for the Lpd-**TDI-13537** complex crystal are shown in Table S3.

The structure of the Lpd-**TDI-13537** complex was solved by molecular replacement (MR) using the program Phaser based on a previously determined structure of Lpd (PDB: 7KMY) as a search model with all heteroatoms removed.^38^ The final model was refined using the Phenix suite.^39^ Refinement statistics are presented in Table S3.

In the refined structure of the Lpd-**TDI-13537** complex, additional electron density was seen near the NADH binding pocket. However, as the density was not fully compatible with either **TDI-13537** or NADH, this pocket was modeled with solvent molecules in the final model.

Atomic coordinates and structure factors were deposited in the Protein Data Bank with accession code 8U0Q.

## Supporting Information Available

Additional details on probes used in the GPU Shape screen, analysis of virtual screen outcome for scores, diversity of the 88 experimentally tested compounds, and hit enrichment; pre-existing SAR for the sulfonamide series; SPR data for **TDI-13537**; X-ray crystallography data collection and refinement statistics for the Lpd-**TDI-13537** co-crystal; details on chemical synthesis and compound purity for **TDI-13537** and **2** (PDF)

## ABBREVIATIONS

Lpd: lipoamide dehydrogenase
TB: tuberculosis
PDH: pyruvate dehydrogenase
MIC: minimum inhibitory concentration
GPU: graphics processing unit
ABFEP: absolute binding free energy perturbation
SAR: structure–activity relationship
