# Supplementary material for: Shape-Based Virtual Screening of a Billion-Compound Library Identifies Mycobacterial Lipoamide Dehydrogenase Inhibitors
Source: ACS Bio Med Chem Au. 2023 Sep 8;3(6):507–15. doi: 10.1021/acsbiomedchemau.3c00046 (PMC10739260; doi:10.1021/acsbiomedchemau.3c00046)
Supplement: Supplementary file 1 — bg3c00046_si_001.pdf [file bg3c00046_si_001.pdf]

**Supplementary Information**

# Shape-Based Virtual Screening of a Billion-Compound Library Identifies Mycobacterial Lipoamide Dehydrogenase Inhibitors

*Mayako Michino[1,*,†], Alexandre Beautrait[2,†], Nicholas A. Boyles[2], Aparna Nadupalli[3], Alexey Dementiev[3], Shan Sun[1], John Ginn[1], Leigh Baxt[1], Robert Suto[3], Ruslana Bryk[4], Steven V. Jerome[2], David J. Huggins[1,5,*], Jeremie Vendome[2,*]*

[1]Sanders Tri-Institutional Therapeutics Discovery Institute, 1230 York Avenue, Box 122, New York, NY 10065, USA

[2]Schrödinger, Inc., 1540 Broadway, 24th Floor, New York, NY 10036, USA

[3]Schrödinger, Inc., 12 Michigan Dr, Natick, MA 01760, USA

[4]Department of Microbiology and Immunology, Weill Cornell Medicine, New York, NY 10065, USA

[5]Department of Physiology and Biophysics, Weill Cornell Medicine, New York, NY 10021, USA

[*]Corresponding authors. Email: mmichino@tritdi.org, jeremie.vendome@schrodinger.com, dhuggins@tritdi.org

[†]Authors contributed equally

**Table of Contents**


| | |
|---|---|
| Pages 3 – 7 | **Table S1.** Known actives used as probes in GPU Shape screen |
| Page 8 | **Figure S1.** Scatterplot of GPU Shape Sim score vs Glide SP docking score |
| Page 9 | **Figure S2.** Chemical diversity of 88 tested compounds |
| Page 10 | **Figure S3.** Hit enrichment using Glide SP docking score vs ABFEP rescoring |
| Page 11 | **Table S2.** Pre-existing SAR of sulfonamide series |
| Page 12 | **Figure S4.** SPR data for **TDI-13537** |
| Page 13 | **Table S3.** Crystallography data and refinement statistics for the Lpd-**TDI-13537** complex |
| Pages 14 – 23 | **Synthesis and Characterization of Compound 1(TDI-13537) and Compound 2** |

**Table S1.** Known actives used as probes in GPU Shape screen
A. 2D structure of 27 probes

| Probes | LPD IC50 (μM) | PDH IC50 (μM) | Mtb MIC (μM) |
|---|---|---|---|
| TDI-010352 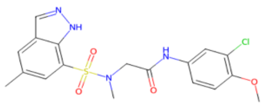 | 0.020 | 0.13 | 25 |
| TDI-010602 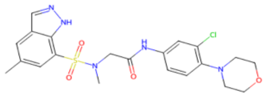 | 0.019 | 0.01 | 13 |
| TDI-010705 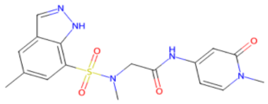 | 0.049 | 0.03 | 4 |
| TDI-011002 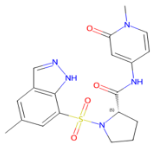 | 0.051 | 0.19 | 25 |
| TDI-011490 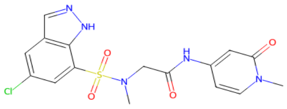 | 0.055 | 0.11 | 13 |
| TDI-011541 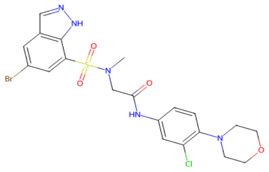 | 0.004 | 0.10 | >25 |
| TDI-011548 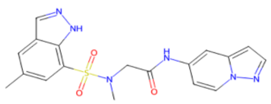 | 0.020 | 0.04 | 13 |

| | | | |
|---|---|---|---|
| TDI-011574 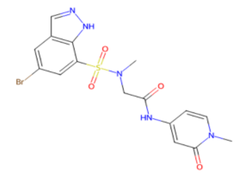 | 0.052 | 0.20 | 6 |
| TDI-011575 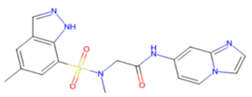 | 0.063 | 0.17 | 25 |
| TDI-011576 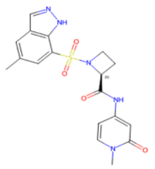 | 0.055 | 0.02 | 6 |
| TDI-011618 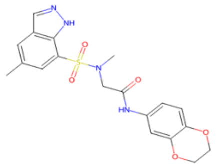 | 0.011 | 0.05 | 25 |
| TDI-011619 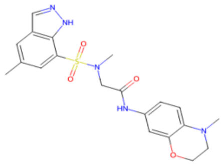 | 0.004 | 0.05 | 3 |
| TDI-011620 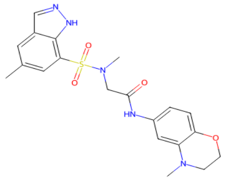 | 0.017 | 0.05 | 13 |
| TDI-011662 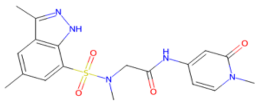 | 0.066 | 0.47 | 50 |
| TDI-011683 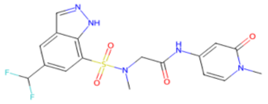 | 0.047 | 0.16 | 6 |

| | | | |
|---|---|---|---|
| TDI-011736 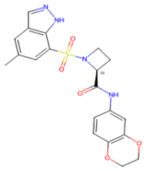 | 0.024 | 0.03 | 25 |
| TDI-011764 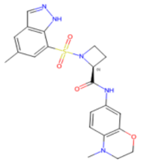 | 0.030 | 0.04 | 9 |
| TDI-011855 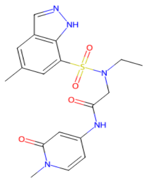 | 0.027 | 0.04 | 6 |
| TDI-012007 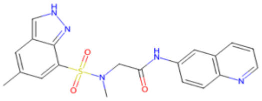 | 0.022 | 0.01 | 3 |
| TDI-012047 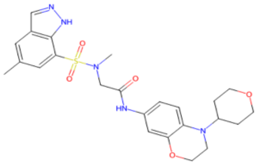 | 0.042 | 0.004 | 1 |
| TDI-012062 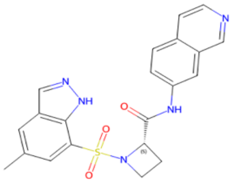 | 0.051 | 0.10 | 13 |
| TDI-012103 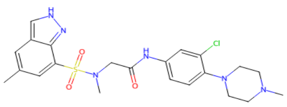 | 0.044 | 0.001 | 2 |
| TDI-012133 | 0.042 | 0.002 | 25 |

| | | | |
|---|---|---|---|
| 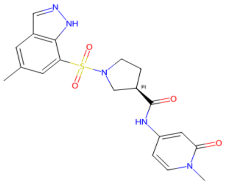 | | | |
| TDI-012138 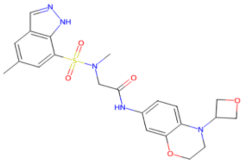 | 0.028 | 0.004 | 2 |
| TDI-012139 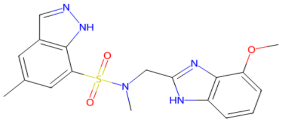 | 0.038 | 0.01 | 50 |
| TDI-012163 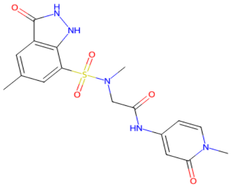 | 0.071 | 0.10 | >100 |
| TDI-012165 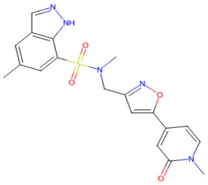 | 0.027 | 0.003 | >100 |

B. Bioactive conformations of the 27 probes superimposed

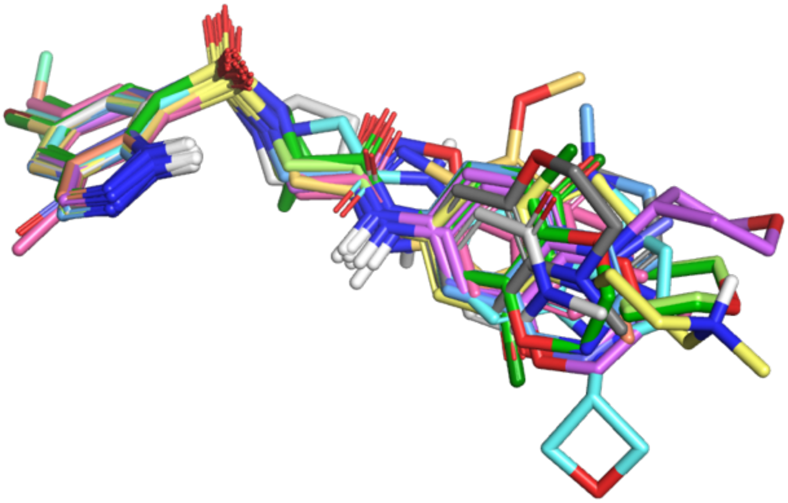

**Figure S1.** Scatterplot of GPU Shape Sim score vs Glide SP docking score. The compounds selected to progress to a visual inspection and voting are either in the lower right corner of the plot (delimited by the red line) or at the very bottom of the plot (delimited by the dashed line).

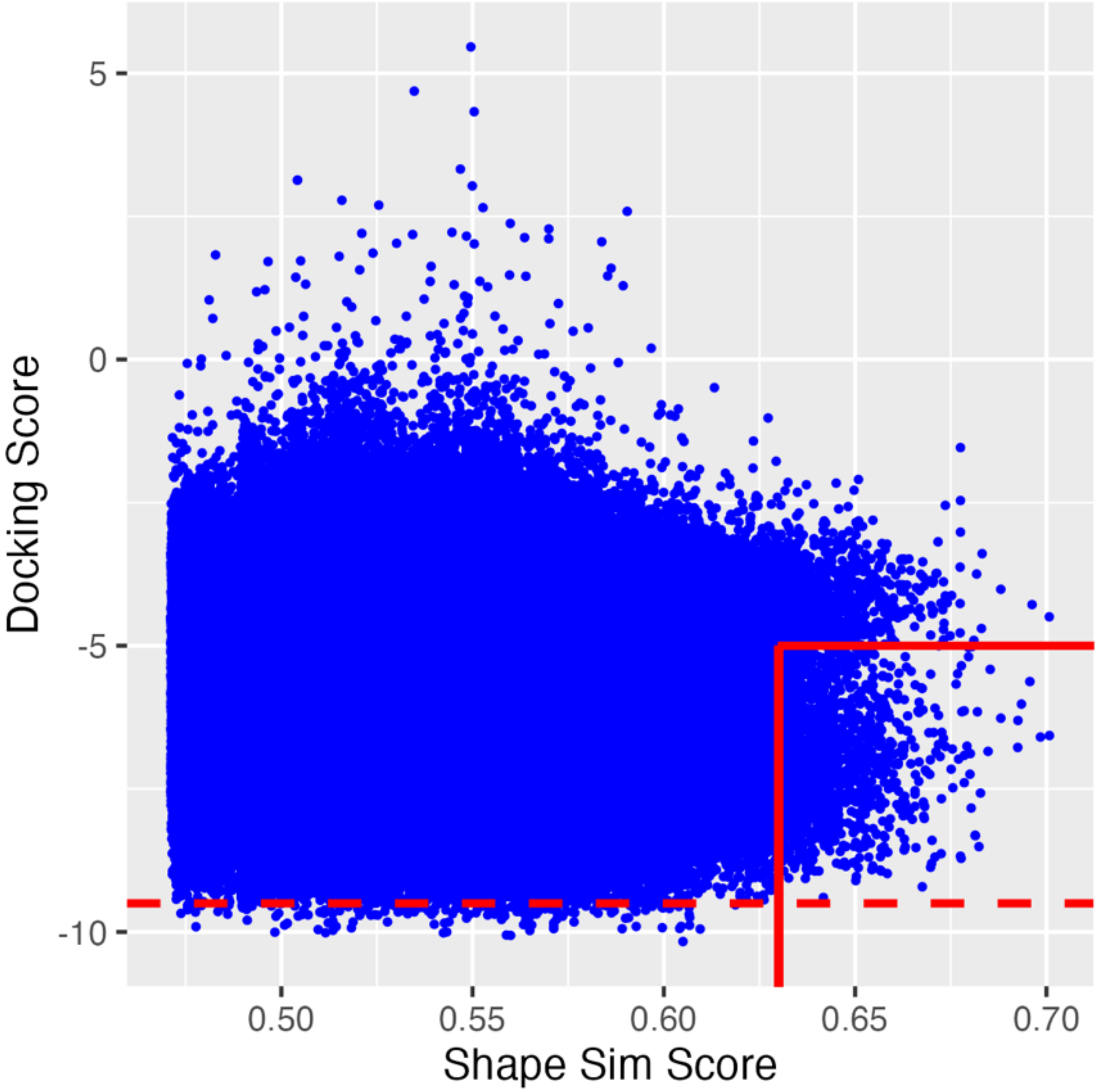

**Figure S2.** Chemical diversity of 88 tested compounds

Heatmap (A) and distribution plots (B) of pairwise Tanimoto similarity scores based on molprint2D fingerprint[1] for the 27 GPU Shape probes and the 88 tested compounds from the virtual screen. Compared to the very high similarity within the set of 27 probes, the 88 tested compounds show more diversity.

A.

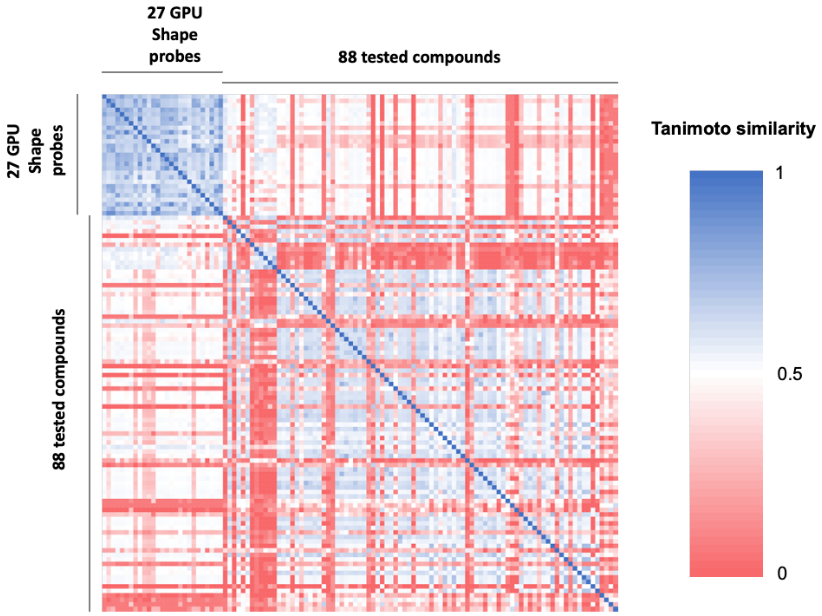


B.

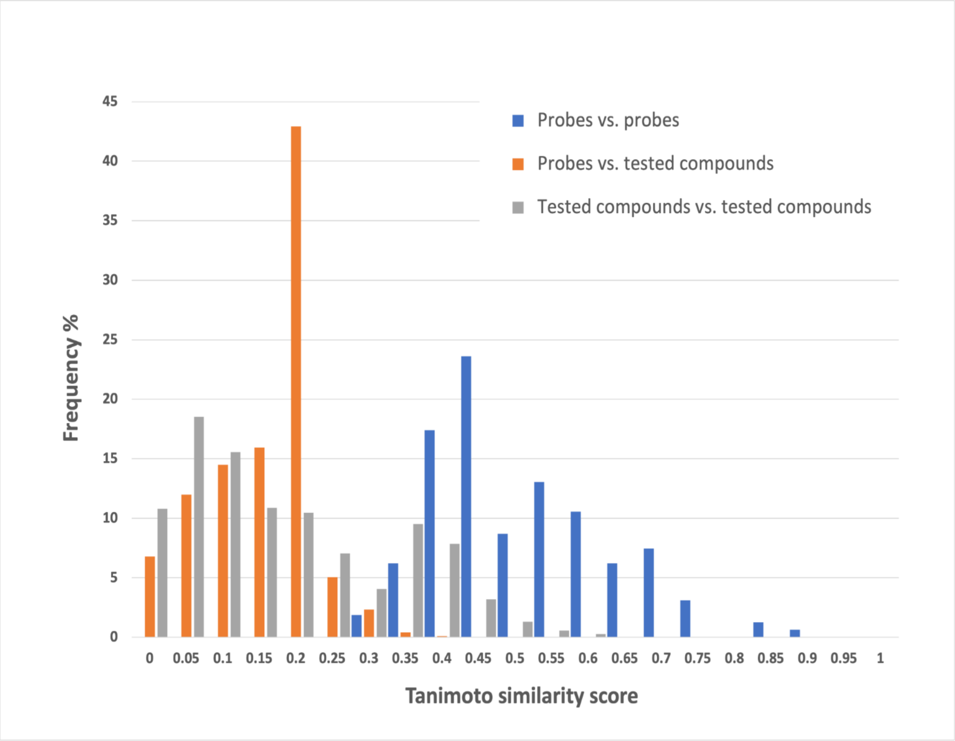

**Figure S3.** Hit enrichment using Glide SP docking score (in orange) vs ABFEP rescoring (in blue).

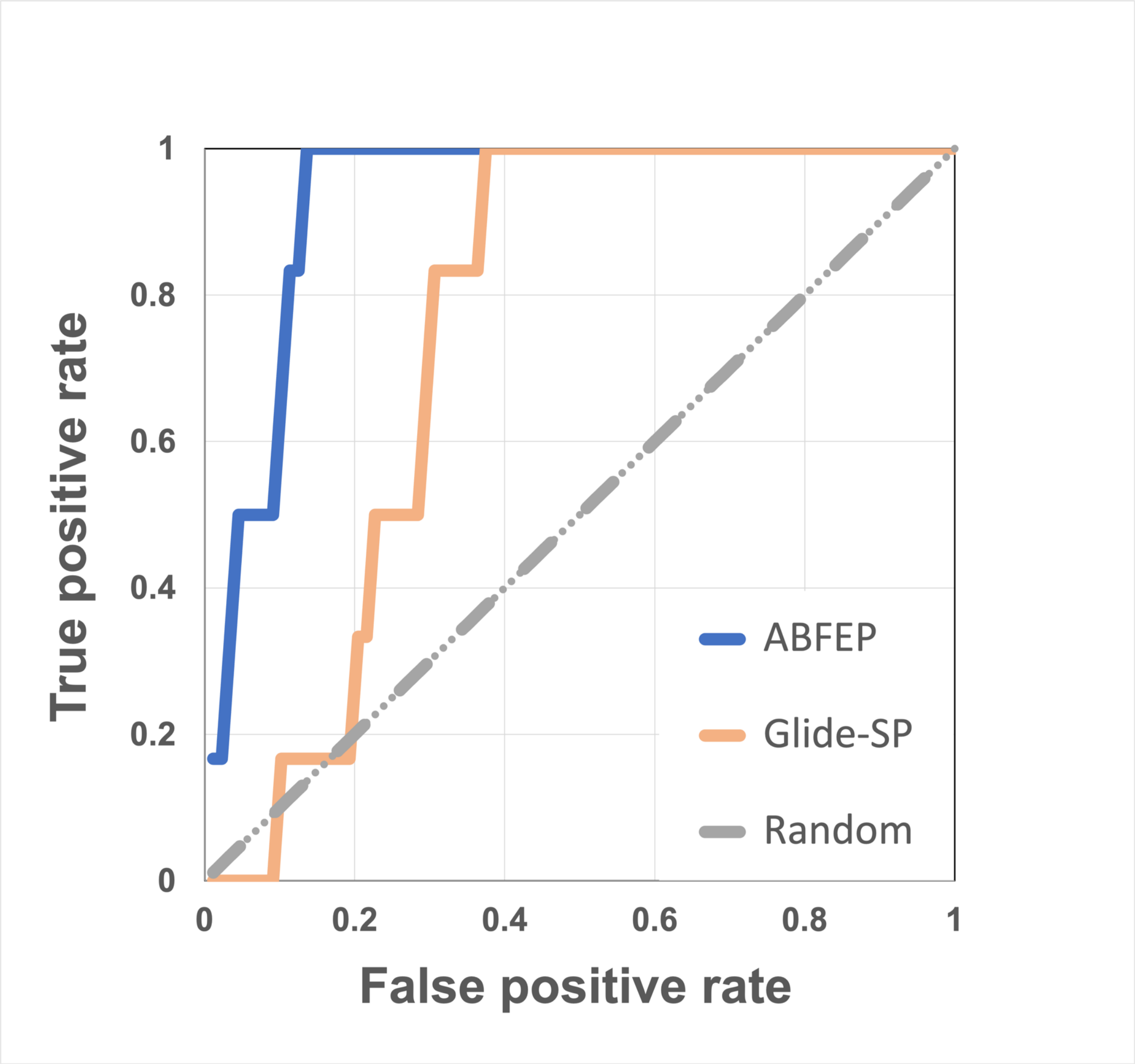

**Table S2.** Pre-existing SAR of sulfonamide series

| Compound ID | Compound Structure | LPD IC50 (μM) |
|---|---|---|
| **S1** | 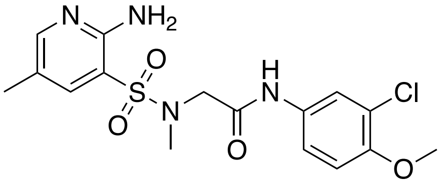 | 0.07 |
| **S2** | 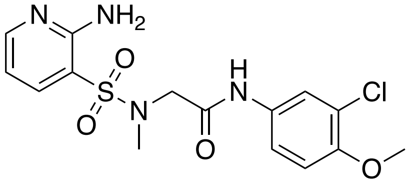 | 5.9 |
| **S3** | 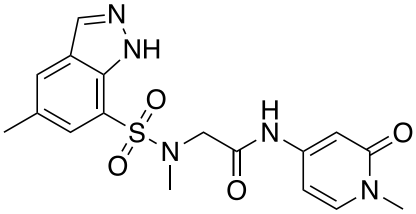 | 0.05 |
| **S4** | 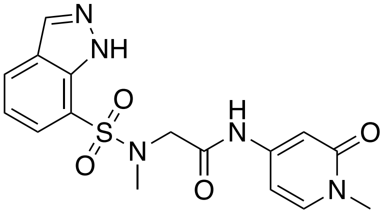 | 1.8 |

**Figure S4.** SPR data for **TDI-13537**

Sensograms showing compound binding to the immobilized protein surfaces. The left image is without NADH, and right image is with NADH.

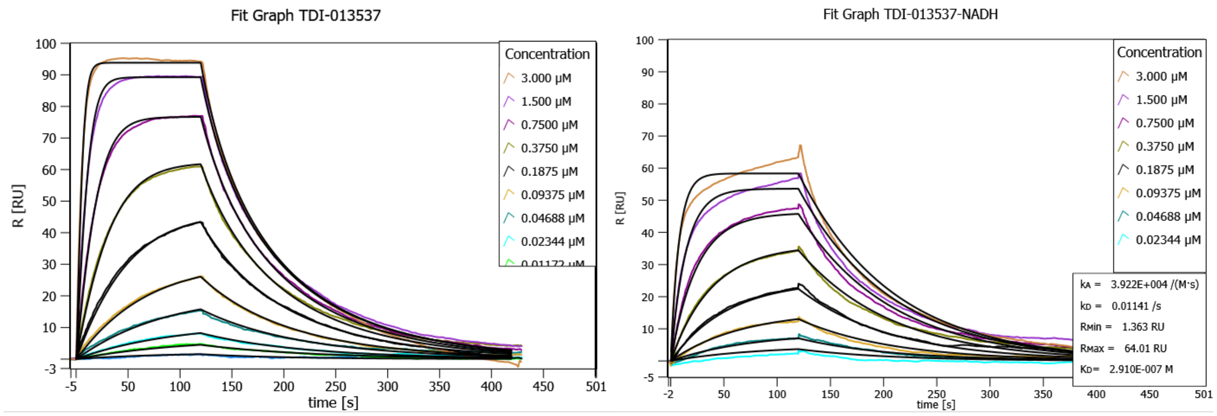


| Analyte | $k_a$ [1/(M·s)] | $k_d$ [1/s] | $R_{max}$ [RU] | $K_D$ [nM] | $\chi^2$ [RU²] |
|---|---|---|---|---|---|
| 013537 | 5.16E+04 | 1.243E-02 | 95 | 241 | 1.5 |
| 013537-NADH | 3.92E+04 | 1.141E-02 | 64 | 291 | 2.32 |

**Table S3.** Crystallography data and refinement statistics for the Lpd-**TDI-13537** complex

| | Lpd-**TDI13537** complex |
|---|---|
| **Wavelength** | 0.95367 |
| **Resolution range** | 44.31 - 1.69 (1.75 - 1.69) |
| **Space group** | P 21 21 21 |
| **Unit cell** | 83.335 97.928 123.806 90 90 90 |
| **Total reflections** | 1361571 (134401) |
| **Unique reflections** | 113737 (11237) |
| **Multiplicity** | 11.2 |
| **Completeness (%)** | 99.95 (99.88) |
| **Mean I/sigma(I)** | 9.5 |
| **Wilson B-factor** | 29.79 |
| **R-meas (%)** | 13.5 |
| **CC1/2 (%)** | 99.5 |
| **Reflections used in refinement** | 113737 (11227) |
| **Reflections used for R-free** | 5677 (570) |
| **R-work** | 0.1698 (0.3614) |
| **R-free** | 0.2063 (0.3923) |
| **Number of non-hydrogen atoms** | 8032 |
| **macromolecules** | 6978 |
| **Ligands** | 498 |
| **Solvent** | 525 |
| **Protein residues** | 928 |
| **RMS(bonds)** | 0.015 |
| **RMS(angles)** | 1.38 |
| **Ramachandran favored (%)** | 96.75 |
| **Ramachandran allowed (%)** | 3.25 |
| **Ramachandran outliers (%)** | 0.00 |
| **Rotamer outliers (%)** | 0.96 |
| **Clashscore** | 5.37 |
| **Average B-factor** | 34.89 |
| **macromolecules** | 32.79 |
| **ligands** | 51.77 |
| **solvent** | 46.06 |

Statistics for the highest-resolution shell are shown in parentheses.

## Synthesis and Characterization of TDI-13537 and Compound 2

**Scheme S1: Synthesis of Intermediate 3**.

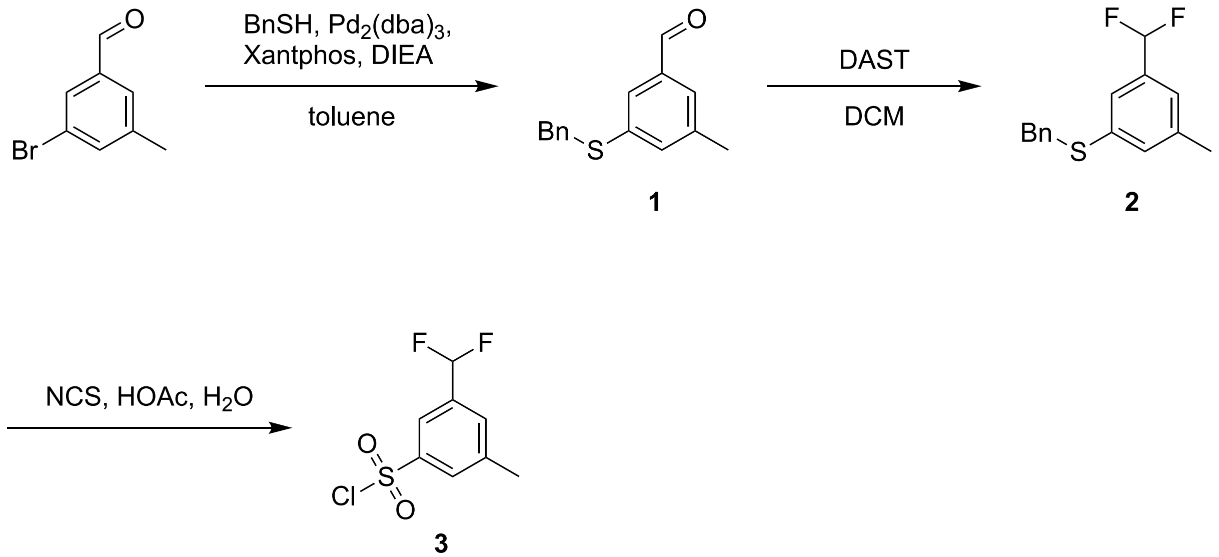


**Synthesis of 3-(benzylthio)-5-methylbenzaldehyde (1).**

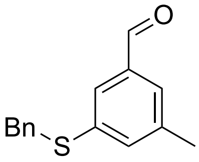

**1**

To a solution of 3-bromo-5-methylbenzaldehyde (2.00 g, 10.1 mmol) in toluene (25 mL) was added N-ethyl-N-isopropylpropan-2-amine (3.90 g, 30.1 mmol), tris(dibenzylideneacetone)dipalladium(0) (0.920 g, 1.00 mmol), (9,9-dimethyl-9H-xanthene-4,5-diyl)bis(diphenylphosphine) (0.581 g, 1.00 mmol) and phenylmethanethiol (1.37 g, 11.1 mmol) at 25 °C. The mixture was heated to 100 °C and stirred for 3 hours. The mixture cooled to room temperature, diluted with water (80 mL), and extracted with ethyl acetate (80 mL*3). The combined organic layers were washed with brine (30 mL*3), dried over anhydrous sodium sulfate, filtered, and the filtrate was concentrated under reduce pressure. The residue was purified by silica gel column chromatography using a gradient of 20 : 1 to 8 : 1 **petroleum ether : ethyl acetate.**

The eluent was evaporated under reduced pressure to afford 3-(benzylthio)-5-methylbenzaldehyde (**1**) (2.30 g, 9.49 mmol, 94.5% yield) as a yellow oil.

**$^1$H-NMR of compound 1:** (DMSO-$d_6$, 400 MHz) δ 9.92 (s, 1H), 7.84 - 7.78 (m, 1H), 7.65 (s, 1H), 7.49 (d, $J$ = 1.6 Hz, 1H), 7.38 (m, 2H), 7.33 - 7.28 (m, 2H), 7.27 - 7.22 (m, 1H), 4.32 (s, 2H), 2.34 (s, 3H).

**Synthesis of benzyl(3-(difluoromethyl)-5-methylphenyl)sulfane (2).**

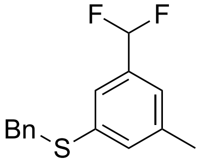


**2**

To a solution of compound **1** (1.00 g, 4.13 mmol) in dichloromethane (10 mL) at 0 °C under nitrogen was added N-ethyl-N-(trifluoro-λ$^4$-sulfanyl)ethanamine (2.99 g, 18.6 mmol). The mixture was warmed to 25 °C and stirred for 1 hour. The mixture was diluted with water (10 mL) and extracted with ethyl acetate (20 mL*3). The combined organic layers were washed with brine (15 mL*3), dried over anhydrous sodium sulfate, filtered, and the filtrate was concentrated under reduce pressure. The residue was purified by silica gel column chromatography using a gradient of 30 : 1 to 5 : 1 petroleum ether : ethyl acetate. The eluent was evaporated under reduced pressure to afford benzyl(3-(difluoromethyl)-5-methylphenyl)sulfane (**2**) (0.250 g, 0.946 mmol, 22.9% yield) as a colorless oil.

**$^1$H-NMR of compound 2:** (DMSO-$d_6$, 400 MHz) δ 7.39 - 7.21 (m, 7H), 7.17 (s, 1H), 6.93 (t, $J$ = 52.0 Hz, 1H), 4.28 (s, 2H), 2.31 (s, 3H)

**Synthesis of 3-(difluoromethyl)-5-methylbenzene-1-sulfonyl chloride (3).**

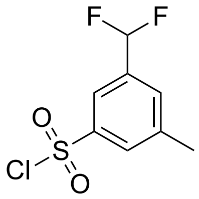

**3**

To a solution of compound **2** (0.100 g, 0.378 mmol) in a mixture of acetic acid (1 mL) and water (0.3 mL) at 0 °C was added 1-chloropyrrolidine-2,5-dione (0.152 g, 1.13 mmol). The mixture was warmed to 25 °C and stirred for 1 hour. The mixture was diluted with water (5mL) and extracted with ethyl acetate (10 mL*3). The combined organic layers were washed with brine (5 mL*2), dried over anhydrous sodium sulfate, filtered, and the filtrate was concentrated under reduce pressure to afford 3-(difluoromethyl)-5-methylbenzene-1-sulfonyl chloride (**3**) (0.080 g, crude) as a yellow oil. No further purification was performed.

**$^1$H-NMR of compound 3:** ($CDCl_3$, 400 MHz) δ = 7.89 (d, *J* = 9.2 Hz, 2H), 7.60 (s, 1H), 6.62 (t, *J* = 56.0 Hz, 1H), 2.47 (s, 3H)

**Scheme S2: Synthesis of Intermediate TDI-013537**.

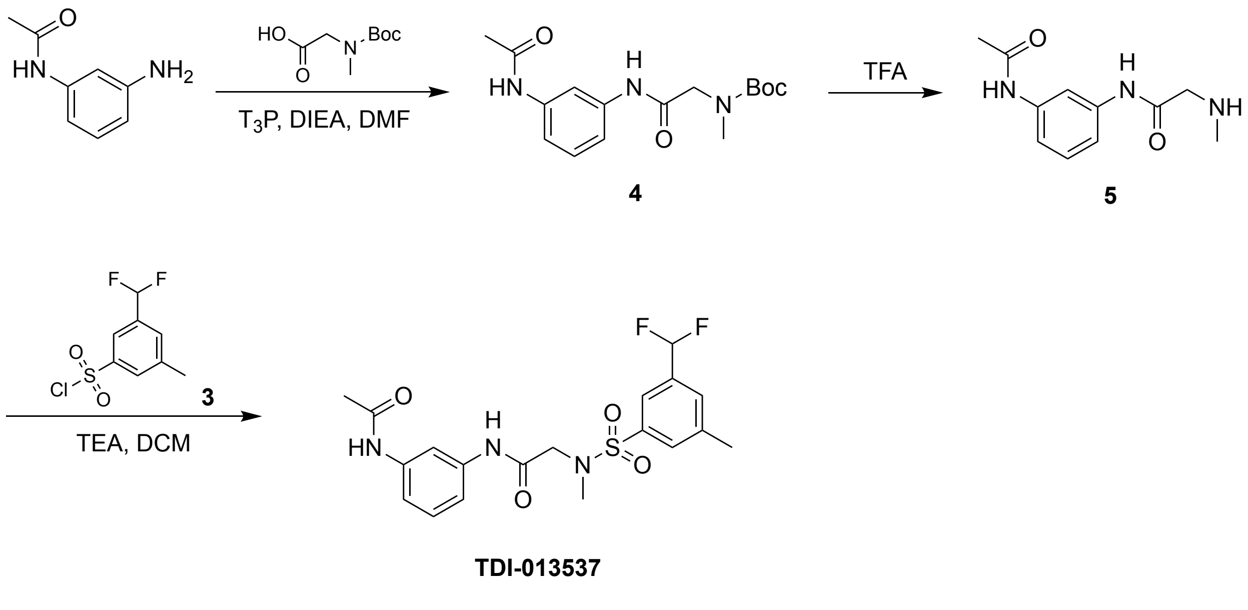


**Synthesis of tert-butyl (2-((3-acetamidophenyl)amino)-2-oxoethyl)(methyl)carbamate (4).**

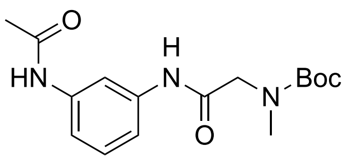

**4**

To a solution of 2-[tert-butoxycarbonyl(methyl)amino]acetic acid (1.26 g, 6.66 mmol) in dimethylformamide (10 mL) at 25°C was added *N*-ethyl-*N*-isopropylpropan-2-amine (1.72 g, 13.3 mmol) and dipropyldiphosphonic acid (6.36 g, 9.99 mmol). The mixture was stirred at 25 °C for 2 hours then *N*-(3-aminophenyl)acetamide (1.00 g, 6.66 mmol) was added. The mixture was warmed to 50 °C and stirred for 12 hours. The mixture was cooled to room temperature, diluted with water (50 mL), and extracted with ethyl acetate (50 mL*3). The combined organic layers were washed with brine (30 mL*3), dried over anhydrous sodium sulfate, filtered, and the filtrate was concentrated under reduce pressure. The residue was purified by silica gel column chromatography using a gradient of 5 : 1 to 1 : 1 **petroleum ether : ethyl acetate.** The eluent was evaporated under reduced pressure to afford tert-butyl (2-((3-acetamidophenyl)amino)-2-oxoethyl)(methyl)carbamate (**4**) (1.20 g, 3.73 mmol, 56.1% yield) as a yellow oil.

**LCMS of compound 4:** RT = 0.366 min, m/z: 322.3 $[M+H]^+$ (96% @ 254nm)

**$^1$H-NMR of compound 4:** ($CDCl_3$, 400 MHz) δ = 8.93 (d, *J* = 2.4 Hz, 1H), 8.75 (br. s, 1H), 7.67 (br. s, 1H), 7.29 - 7.05 (m, 3H), 3.88 (s, 2H), 2.05 - 2.02 (m, 3H), 1.99 - 1.83 (m, 3H), 1.36 (br. s, 9H)

**Synthesis of tert-butyl (2-((3-acetamidophenyl)amino)-2-oxoethyl)(methyl)carbamate (5).**

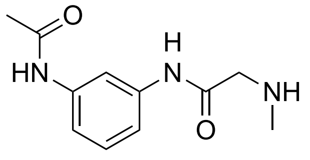

**5**

To a solution of compound **4** (0.500 g, 1.56 mmol) in dichloromethane (5 mL) at 25 °C was added 2,2,2-trifluoroacetic acid (2.31 g, 20.3 mmol). The mixture was stirred at 25 °C for 1 hour then concentrated under reduced pressure. The residue was purified by preparative reverse phase HPLC (column: Phenomenex Gemini 150*25 mm*10 um) using a mobile phase of 1%-30% water

(w/0.05% $NH_3H_2O$) : Acetonirile. The eluent was evaporated under reduced pressure to afford *N*-(3-acetamidophenyl)-2-(methylamino)acetamide (**5**) (0.100 g, 0.452 mmol, 29.1% yield) as a yellow oil.

**LCMS of compound 5:** RT = 0.583 min, m/z: 222.2 $[M+H]^+$ (> 99% @ 254nm)

**$^1$H-NMR of compound 5:** (DMSO-$d_6$, 400 MHz) δ = 8.81 (d, *J* = 4.4 Hz, 2H), 7.96 (d, *J* = 11.6 Hz, 2H), 7.36 - 7.30 (m, 1H), 7.28 - 7.18 (m, 2H), 3.91 (t, *J* = 5.6 Hz, 2H), 2.73 (s, 3H), 2.04 (s, 3H)

**Synthesis of *N*-(3-acetamidophenyl)-2-(3-(difluoromethyl)-N,5-dimethylphenylsulfonamido)acetamide (TDI-013537).**

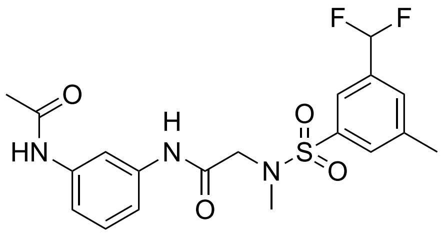

**TDI-013537**

To a solution of compound **5** (0.100 g, 0.452 mmol) in dimethylformamide (2 mL) at 0 °C was added triethylamine (60.4 g, 0.597 mmol) and compound **3** (0.072 g, 0.298 mmol). The mixture was warmed to 25 °C and stirred for 0.5 hour. The solution was diluted with water (10 mL) and the aqueous phase was extracted with ethyl acetate (10 mL*3). The combined organic layers were washed with brine (5 mL*2), dried over anhydrous sodium sulfate, filtered, and the filtrate was concentrated under reduce pressure. The residue was purified by preparative reverse phase HPLC (column: Welch Xtimate $C_{18}$ 150*25 mm*5 um) using a gradient of 28% to 58% water (w/ 0.05%HCl) : acetonitrile. The eluent was removed under reduced pressure to afford *N*-(3-acetamidophenyl)-2-(3-(difluoromethyl)-N,5-dimethylphenylsulfonamido)acetamide (TDI-013537) (0.031 g, 0.073 mmol, 16.2% yield) as a white solid.

**LCMS of TDI-013537:** RT = 2.031 minutes, m/z: 426.1 $[M+H]^+$ (100% @ 254 nM)

**$^1$H-NMR of TDI-013537**: ($CD_3OD$, 400 MHz) δ = 7.86 - 7.73 (m, 3H), 7.66 (s, 1H), 7.32 - 7.19 (m, 3H), 6.83 (t, *J* = 55.6 Hz, 1H), 3.98 (s, 2H), 2.93 (s, 3H), 2.48 (s, 3H), 2.11 (s, 3H)

**Scheme S3: Synthesis of Intermediate 7**.

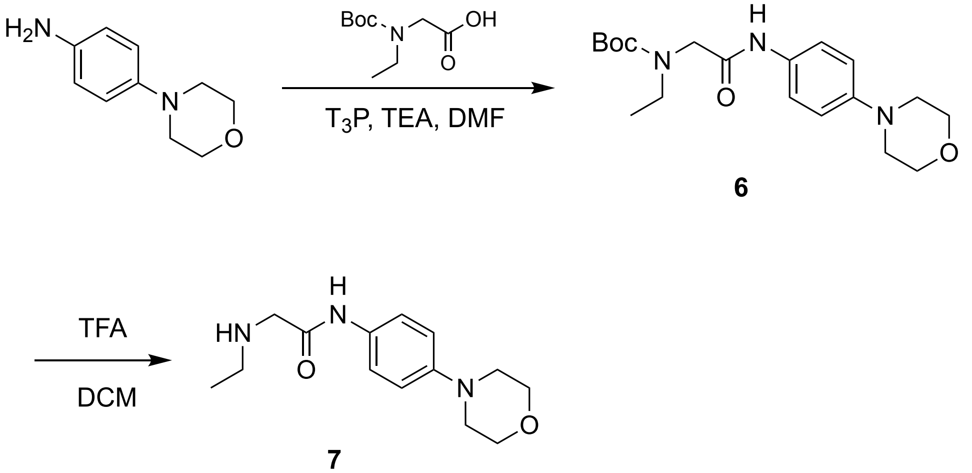


**6**

**7**

**Synthesis of *tert*-butyl ethyl(2-((4-morpholinophenyl)amino)-2-oxoethyl)carbamate (6).**

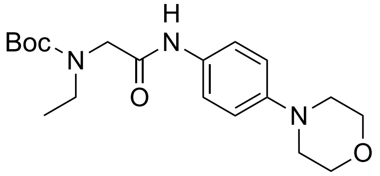

**6**

To a solution of 4-morpholinoaniline (2.00 g, 11.2 mmol) and 2-((tert-butoxycarbonyl)(ethyl)amino)acetic acid (2.28 g, 11.2 mmol) in dimethyl formamide (40 mL) was added $T_3P$ (7.14 g, 11.2 mmol, 50% solution in ethyl acetate) followed by diisopropylethylamine (4.35 g, 33.7 mmol). The mixture was heated to 50 °C and stirred for 12 hours. The mixture was cooled to room temperature and poured into ice-water (100 mL). The mixture was extracted with ethyl acetate (75 mL*3) and the combined organic layers were washed with brine (75 mL*2), dried over anhydrous sodium sulfate, filtered, and the filtrate was concentrated under reduced pressure to afford *tert*-butyl ethyl(2-((4-morpholinophenyl)amino)-2-oxoethyl)carbamate (**6**) (3.50 g, crude) as a brown solid. No further purification was performed.

**LCMS of compound 6:** RT = 0.374 minutes, *m/z* 364.2 $[M+H]^+$ (> 99% @ 254nm)

**Synthesis of tert-butyl ethyl(2-((4-morpholinophenyl)amino)-2-oxoethyl)carbamate (7).**

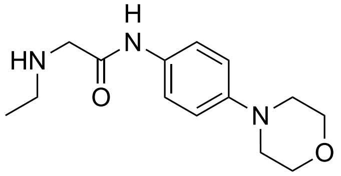


**7**

A solution of compound **9** (3.50 g, 9.63 mmol) in a mixture of trifluoroacetic acid (4 mL) and dichloromethane (20 mL) was stirred at 20 °C for 1 hour . The mixture was concentrated under reduced pressure and the residue purified by reversed-phase flash chromatography using an eluent of water : acetonitrile with 0.1% $NH_3$•$H_2O$. The eluent was removed under reduced pressure to afford 2-(ethylamino)-*N*-(4-morpholinophenyl)acetamide (**6**) (4.00 g).

**LCMS of 7:** RT = 0.766 minutes, *m/z* 264.3 $[M+H]^+$ (94% @ 254nm)

**Scheme S4: Synthesis of Compound 2**.

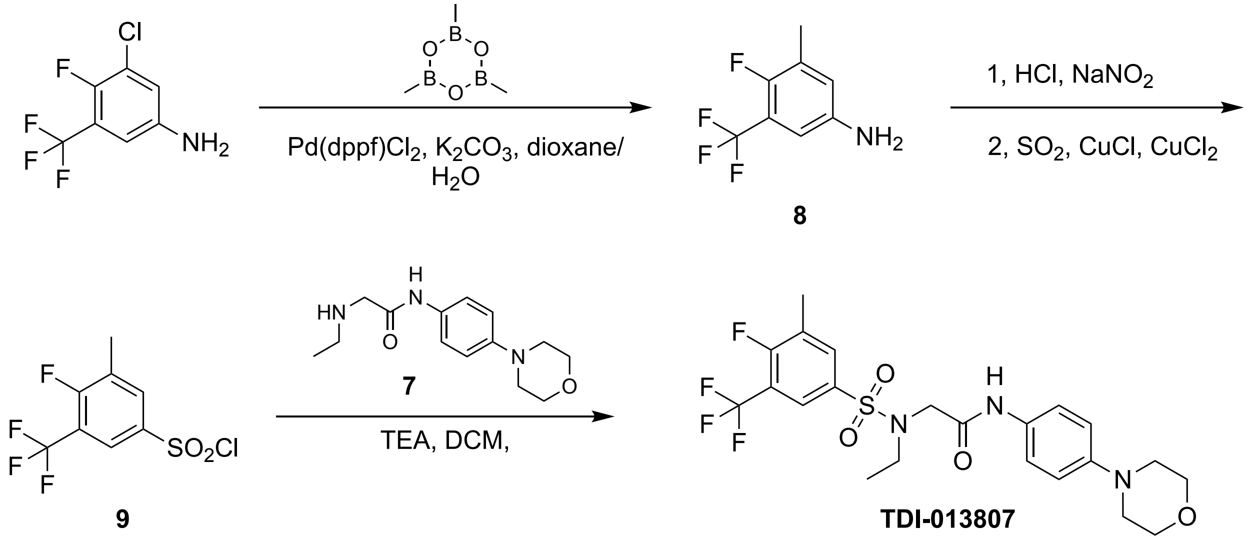


**Synthesis of 4-fluoro-3-methyl-5-(trifluoromethyl) aniline (8).**

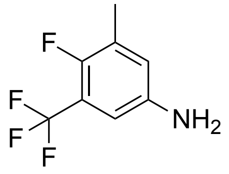


**8**

A mixture of 3-chloro-4-fluoro-5-(trifluoromethyl) aniline (0.500 g, 2.34 mmol), 2,4,6-trimethyl-1,3,5,2,4,6-trioxatriborinane (2.94 g, 11.7 mmol, 50%), cyclopenta-2,4-dien-1-yl(diphenyl)phosphane iron(II) dichloropalladium (0.171 g, 0.234 mmol), and potassium carbonate (0.970 g, 7.02 mmol) were placed up into a microwave tube. Dioxane (10 mL) and water (2 mL) were added and the mixture was placed under nitrogen. The sealed tube was heated at 120 °C for 4 hours under microwave irradiation. The cooled mixture was filtered and the filtrate was poured into 50 mL water and extracted with ethyl acetate (50 mL*2). The combined organic layers were washed with brine (35 mL*2), dried over anhydrous sodium sulfate, filtered, and the filtrate was concentrated under reduced pressure. The residue was purified by preparation TLC (Silica gel, petroleum ether/ethyl acetate = 3:1) to afford 4-fluoro-3-methyl-5-(trifluoromethyl) aniline (**8**) (0.350 g, 1.67 mmol, 71.2% yield) as a white solid.

**LCMS of compound 8:** RT = 0.294 min, *m/z* 194.1 $[M+H]^+$ (92% @ 254nm)

**$^1$H-NMR of compound 8:** ($CDCl_3$, 400 MHz) δ 6.70 - 6.67 (m, 2H), 3.72 - 3.44 (m, 2H), 2.24 (s, 3H).

**Synthesis of 4-fluoro-3-methyl-5-(trifluoromethyl) aniline (9).**

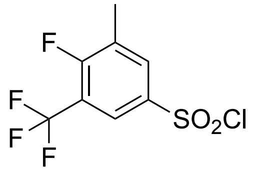


**9**

To a mixture of compound **8** (0.200 g, 1.04 mmol) in acetonitrile (10 mL), hydrochloride acid (1 mL) and water (1 mL) at -5 °C was added an aqueous solution of sodium nitrite (0.093 g, 1.35 mmol). The mixture was stirred at -5 °C for 45 minutes. In a separate flask containing a solution of copper(I) chloride (0.103 g, 0.104 mmol), copper(II) chloride (0.084 g, 0.621 mmol) in acetic acid (1 mL) and water (1 mL) at 0 °C was bubbled sulfur dioxide (15 psi) for 20 minutes. Then the first reaction mixture prepared above was added to this flask. The mixture was warmed to 20 °C and stirred for 30 minutes. After that the mixture was poured in 25 mL of ice-water and extracted with ethyl acetate (25 mL*3). The combined organic layers were washed with brine (25

mL*2), dried over anhydrous sodium sulfate, filtered, and the filtrate was concentrated under reduced pressure. The residue was purified by preparation TLC (Silica gel, petroleum ether/ethyl acetate = 3:1) to afford 4-fluoro-3-methyl-5-(trifluoromethyl) benzene-1-sulfonyl chloride (**9**) (0.130 g, 0.470 mmol, 45.4% yield) as a yellow solid, which was used directly.

**Synthesis of 2-(*N*-ethyl-4-fluoro-3-methyl-5-(trifluoromethyl) phenylsulfonamido)-N-(4-morpholinophenyl) acetamide (Compound 2).**

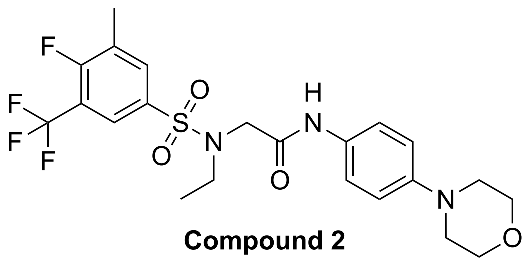


**Compound 2**

To a solution of compound **7** (0.124 g, 0.470 mmol) in dichloromethane (2 mL) was added triethylamine (0.118 g, 1.17 mmol) followed by compound **9** (0.130 g, 0.470 mmol). The mixture was stirred at 20 °C for 1 hour then concentrated under reduce pressure. The residue was purified by preparation reverse phase HPLC (column: Phenomenex Luna $C_{18}$ 100*30 mm*5 um) using a gradient of 45%-75% water (FA) : acetonitrile. The eluent was removed by lyophilization to afford 2-(*N*-ethyl-4-fluoro-3-methyl-5-(trifluoromethyl) phenylsulfonamido)-*N*-(4-morpholinophenyl) acetamide (**Compound 2**) (0.028 g, 0.055 mmol, 11.8 % yield) as a yellow solid.

**LCMS of Compound 2:** RT = 1.462 minutes, *m/z* 504.0 $[M+H]^+$ (100% @ 254nm)

**$^1$H-NMR of Compound 2:** (DMSO-$d_6$, 400 MHz) δ 9.85 (s, 1H), 8.16 (dd, $J_1$ = 2.0 Hz, $J_2$ = 6.8 Hz, 1H), 7.97 (d, $J_1$ = 4.4 Hz, 1H), 7.35 (d, $J$ = 8.8 Hz, 2H), 6.88 (d, $J$ = 8.8 Hz, 2H), 4.09 (s, 2H), 3.73 - 3.71 (m, 4H), 3.35 - 3.33 (m, 2H), 3.04 - 3.02 (m, 4H), 2.35 (d, $J$ = 2.0 Hz, 3H), 1.10 (t, $J$ = 6.8 Hz, 3H).

(1) Duan, J.; Dixon, S. L.; Lowrie, J. F.; Sherman, W. Analysis and comparison of 2D fingerprints: insights into database screening performance using eight fingerprint methods. *J Mol Graph Model* **2010**, *29* (2), 157-170. DOI: 10.1016/j.jmgm.2010.05.008 From NLM Medline.
